# The Safety Attitudes of Senior Managers in the Chinese Coal Industry

**DOI:** 10.3390/ijerph13111147

**Published:** 2016-11-17

**Authors:** Jiangshi Zhang, Na Chen, Gui Fu, Mingwei Yan, Young-Chan Kim

**Affiliations:** 1 School of Resources and Safety Engineering, China University of Mining and Technology, Beijing 100083, China; zjsh0426@163.com (J.Z.); fugui@cumtb.edu.cn (G.F.); yanmingwei1116@163.com (M.Y.); 2 School of Mechanics and Engineering Science, Zhengzhou University, Zhengzhou 450001, China; 3 Department of Civil and Environmental Engineering, University of Michigan, 2350 Hayward St., G.G. Brown Building, Ann Arbor, MI 48109, USA; yyoungchani@gmail.com

**Keywords:** Chinese coal industry, senior manager, attitudes towards safety, safety concepts, questionnaire

## Abstract

*Introduction:* Senior managers’ attitudes towards safety are very important regarding the safety practices in an organization. The study is to describe the current situation of senior managers′ attitudes towards safety in the Chinese coal industry. *Method*: We evaluated the changing trends as well as the reasons for these changes in the Chinese coal industry in 2009 and in 2014 with 168 senior manager samples from large Chinese state-owned coal enterprises. Evaluations of 15 safety concepts were performed by means of a questionnaire. *Results and Conclusions*: Results indicate that, in 2014, three concepts were at a very high level (mean > 4.5), and six were at a relatively high level (4.5 > mean > 4.0). Analyses of changing trends revealed that nine concepts improved significantly, while four greatly declined in 2014 compared to those in 2009. The data reported here suggest that the reasons for the significant improvement with respect to the nine concepts include the improvement in social and legal environments, the improvement of the culture of social safety, workers′ safety demands being met, and scientific and technical advances in the coal industry. The decline of the four concepts seemed to be caused by a poor awareness of managers in the coal industry that safety creates economic benefits, insufficient information on safety, inadequate attention to the development of a safety culture and safety management methods, and safety organizations and workers′ unions not playing their role effectively. *Practical Applications*: We therefore recommend strengthening the evidence that safety creates economic benefits, providing incentives for employees to encourage their participation in safety management, and paying more attention to the prevention of accidents in coal mines via safety organizations and unions. These results can provide guidelines for workers, industrialists, and government regarding occupational safety in the whole coal industry.

## 1. Introduction

Comprehensive statistics have indicated that human factors (including deliberate violation, management failures, and faulty design) accounted for 97.67% of Chinese coal mine accidents [1]. Numerous studies held the view that one way to avoid incidents related to human factors was to improve managers’ attitudes towards safety issues [2]. Therefore it is important to determine managers’ views regarding safety in the coal industry. The main objectives of this study were to reveal senior managers’ attitudes towards safety, their changing trends over time and the fundamental reasons for them, and countermeasures to reduce accidents. For this purpose, we conducted surveys in several large state-owned coal enterprises in China in 2009 and in 2014. A quantitative analysis was performed using the Safety Culture Online Analysis Program (SCAP), which was developed by our research team. The results presented here can provide guidelines for workers, industrialists, and government regarding occupational safety in the Chinese coal industry.

Individual attitudes towards safety are an important factor in the success of creating a safe workplace climate. These attitudes reflect individually constructed beliefs and emotions with regard to safety policies, procedures, and practices [3,4], including personal safety commitments and responsibility. Studies have shown that individual attitudes and behaviors are closely related. It has also been shown that attitudes towards safety can significantly predict risk-taking behavior negatively [5,6]. The attitudes of individuals working at managerial posts not only affect their own decision making but also affect, directly or indirectly, the attitudes and behaviors of staff working under them.

Senior managers’ attitudes towards safety are very important regarding the safety practices in an organization, as they participate in discussions, make decisions, coordinate the entire organization, and thus have great decision-making power and control. A review of the literature shows that managers involved in safety play a key role in safety climate, safety behavior, safety performance, and outcomes, but the research about their individual attitudes and its impact on safety is rare.

The effects that managers involved in safety can have on the safety of a given workplace has been examined in various studies [3,7,8,9]. In addition, the importance of managers′ styles has also been studied in different organizational settings that create a positive work climate and positive safety outcomes [10,11]. Several other studies have also been conducted on the importance of managers in safety outcomes [9], on the role of managers in creating a climate that encourages safety behavior [2,10,12,13,14,15], and the relationship among safety managers, safety climate, safety performance, and safety outcomes [16,17,18,19,20,21,22]. In addition, O′Dea and Flin [23] explored the factors relevant to positive safety outcomes (incidents, accidents, and near-misses) and narrowed them down to four basic factors, namely senior management factors, management factors, supervisory factors, and employee factors. The result was that safety simply cannot be realized if senior managers’ attitudes towards safety are inadequate. Senior management factors included attitude towards safety, manager style, and trust.

It is important to describe here that most of the research on managers involved in safety has been conducted in various industrial sectors, such as manufacturing, the chemical industry, and construction [10,15,24,25,26], but rarely in the coal industry. Therefore, empirical research on senior managers′ attitudes towards safety in the coal industry is quite necessary in order to reduce accidents and obtain positive safety outcomes.

## 2. Methods

In 1980, Zohar began to study the structures and mechanisms of attitudes towards safety [27]. Years later, Guldenmund carried out related research [28]. However, the measurement content and questions have not reached an agreement. Measuring methods in these studies commonly include scales, especially the Likert scale. Stewart, from Canada, made a safety culture element table, which is one of the methods worth recommending [29]. He studied ten enterprises with advanced safety performance and five with poor safety performance. He used 25 measuring questions, which consisted of 25 element indexes, which could be divided into three groups: basic safety concepts, main safety methods, and basic working practices. In China, the related work in the national model safety culture enterprises began in 2010, and the main national safety culture standards all involved senior managers′ attitudes towards safety, such as “Directives for Developing Enterprise Safety Culture” (AQ/T9004-2008) and “Assessment Standards of Enterprise Safety Culture Developing” (AQ/T 9005-2008). According to these related Chinese safety culture developing standards, and considering the Chinese coal industry′s characteristics and senior managers′ job responsibility (see Section 2.2.1), using the safety culture element table (including 25 indexes) developed by Stewart J. M. [29] for reference, the scale and tools of safety culture measurement in our research team were developed [30]. The scale was modified and simplified adaptively based on Stewart’s 25 safety culture element indexes [29], and finally was set to 15 safety concept indexes (see Table 1), which is only applicable to senior managers in the Chinese coal industry. The questionnaire also had 15 questions and used the Likert scale. These 15 concept indexes, coming from enterprise observations, were validated by enterprises′ actual data and used for the evaluation on the improvement of safety performance. This paper also uses these 15 concept indexes and analyzes the current status and changing trends of senior managers’ attitudes towards safety in 2009 and in 2014.

### 2.1. Fifteen Important Safety Concept Indexes

The focus here is on analyzing fifteen important safety concept indexes closely related to senior managers′ attitudes towards safety, which have great impacts on enterprise safety. The meanings of the fifteen concept indexes are listed in Table 1.

### 2.2. Sample and Procedures

#### 2.2.1. Sample

Using the opportunities of senior managers′ safety qualification training, this study obtained a total of 168 valid sample data in 2009 and in 2014, and each stage collected 84 valid samples of senior managers. Before data collection, a brief description and content explanation was given to the sampled personnel, confirming that respondents were anonymous and data acquisition was accurate.

It is worth mentioning that the surveys in both stages were exactly the same, and the data were well representative of the coal industry. All subjects were senior managers from Chinese large state-owned coal mine enterprises. The senior managers here included the chairman of the board, the general manager, the work safety administration chief, the chief engineer, etc. in a single enterprise. The chairman of the board is the leader of the board of directors, has executive power, is responsible for the organization and its coordination, and leads the enterprise in making sound strategic decisions. The general manager is the top manager in charge of the entire company, responsible for the company′s daily business operations, and reports to the board of directors. The work safety administration chief is the director general of the State/Local Administration of Work Safety, who is in charge of the production safety and related safety policies and regulations. The chief engineer is mainly responsible for safety and engineering technology. Subjects come from 11 provinces and 48 different coal enterprises in China, including Shenhua Coal Industry Co., Ltd., Hebei Jizhong Energy Group Co., Ltd., Shandong Yanzhou Mining Group Co., Ltd., Shanxi Huozhou Coal and Electricity Co., Ltd., Shanxi Taiyuan Coal Gasification Group Co., Ltd., Jincheng Anthracite Mining (Group) Co., Ltd., Shanxi Fenxi Mining Group Company, Shanxi Datong Coal Mine Group Co., Ltd., etc. The sample data is highly representative and accurately reflects Chinese senior managers′ attitudes towards safety.

#### 2.2.2. Procedures

The site was evaluated based on the 15 safety concept indexes mentioned above by the questionnaire and a five-point Likert scale. Comparative analyses were carried out on the evaluation scores at both stages. We developed the questionnaire to determine individuals′ perceptions of enterprise safety, providing the foundation for promoting a safety culture and creating a world-class safety routine. The scale we used here was suitable for senior managers only. In the process of evaluation, respondents were able to answer questions together with their personal understandings. Moreover, the response to each question was divided into five levels, which were respectively given a score of 1, 0.8, 0.6, 0.4, and 0.2. Some questions were scored reversely, and total scores were finally obtained.

## 3. Results and Analysis

### 3.1. Data Statistics Analysis and Comparision

Scale data processing is usually twofold. First, the reliability and validity is tested, extracting the main factor and exploring the structural relationship via software, such as lisrel (Scientific Software International Inc., Skokie, IL, USA), which is commonly used. Second, the status and change in attitude towards safety is evaluated using direct summation, mean, *t*-tests, etc. for statistics comparison. Stewart J.M. [29] also used the latter method. Here, we measure the current status and changing trend in senior managers′ attitudes towards safety, so the data is processed similarly using the latter method.

The mean, standard deviation, and *t*-test values were calculated for a comparative analysis of all 15 concepts at both stages (2009 and 2014) (Table 2). The *t*-test is mainly used for a small sample (sample size is less than 30) to test the difference between two average values, and it uses the *t*-distribution theory to infer the probability that a difference occurs and to determine whether the two average values are significantly different or not. If a certain evaluation concept has no significant effect, then the *t*-value will probably be zero.

### 3.2. Current Status Analysis (2014)

The comparative analysis of the 15 safety evaluation concepts presented here shows (Table 2) that there are only 3 concepts at a very high level (mean > 4.5) in the second stage (2014). These are the degree of safety merged into enterprise management (4.9275), the demands for safety training (4.8551), and the recognition of the work of the safety department (4.8939). Therefore, it is evident that a high importance is given to safety management systems, safety professional institutions, safety working groups, and safety training by the managers in the coal industry.

In addition, there are six items at a relatively high level (4.5 > mean > 4.0) in the following concepts: understanding that casualties are preventable (4.2319), understanding that safety creates economic benefits (4.0435), the opinion that safety mainly depends on safety awareness (4.2029), understanding the role of safety management systems (4.1014), cognition of safety performance and human resources (4.3206), and emergency response capability (4.0876). From these data, it can be inferred that senior managers also pay great attention to the selection of safety staff, accident prevention, the concept that safety creates economic benefits, and the safety management system.

However, it appears from the data that relatively little attention is given to the following safety issues by senior managers: the perception of the relative importance of safety (3.8986), understanding the responsibility system of work safety (3.9638), opinion on safety investment (3.2059), the role of safety regulation (3.5036), managers′ responsibility degree for safety (3.2993), and the mastery of safety methods (3.5556). The reason may be that, although China has emphasized “safety first” and many enterprises are currently carrying out a “safety veto,” enterprises still value economic benefits more than safety performance and may not increase investment in safety. Moreover, as far as managers are concerned, the improvement of economic benefits is beneficial in terms of promotion, rewards, etc., while safety performance does not benefit them as much. Moreover, the concepts of the role of safety regulation (3.5036) and of the mastery of safety methods (3.5556) were also scored at a low level; therefore, enterprises should strengthen them.

### 3.3. Changing Trend Analysis (2014 vs. 2009)

It is believed that managers′ perceptions of safety issues have improved overall in 2014 compared with 2009. There are nine concepts in 2014 significantly higher than in 2009: understanding that casualties are preventable (*t* = 1.554), the degree of safety merged into enterprise management (*t* = 1.388), understanding the responsibility system of work safety (*t* = 2.028), opinion on safety investment (*t* = 5.645), the role of safety regulation (*t* = 9.799), understanding the role of safety management systems (*t* = 4.660), cognition of safety performance and human resources (*t* = 8.297), recognition of the work of the safety department (*t* = 19.485), and emergency response capability (*t* = 4.935).

On the other hand, the level of the following four concepts significantly decreased from 2009 to 2014: understanding that safety creates economic benefits (*t* = −3.494), the opinion that safety mainly depends on safety awareness (*t* = −1.969), managers′ responsibility degree for safety (*t* = −10.045), and the mastery of safety methods (*t* = −9.334). The changing trends in the remaining two concepts—the perception of the relative importance of safety (*t* = 0.968) and the demands for safety training (*t* = −0.818)—are not obvious.

## 4. Discussion

### 4.1. Causes of Some Safety Evaluation Concepts′ Improvement

#### 4.1.1. Improvement of the Social and Legal Environment

The basic pattern of safety work in coal mines stabilized after a series of changes. For example, the State Administration of Coal Mine Safety was founded in January 2000, and the General Office of the State Council issued guidelines in November 2004 for improving the supervision of safety systems in coal mining, establishing the following pattern levels of coal mine safety work: national supervision, local monitoring, and enterprise responsibility. In order to improve occupational safety situation in coal mining enterprises, the State Council upgraded the administration level of the State Administration of Work Safety and appointed the State Administration of Coal Mine Safety to improve monitoring authority and enact power to enforce laws on safety supervision on 23 February 2005.

The strategy of public safety by law was put into practice. In recent years, several laws and regulations on safety have been implemented, such as the Safety Production Law, the Coal Law, the Mine Safety Act, the Law of People′s Republic of China on Prevention and Control of Occupational Diseases, the OSH Legislation, Regulations Concerning Safe Production License, Industrial Insurance Rules, and the Decision by the State Council. In addition, obligations for the safe production systems in coal mines was gradually widened to include accountability for coal mine accidents, particularly stressing that the main responsibility lies with the person in charge of safe production work.

#### 4.1.2. Improvement of the Social Safety Culture

(1) Not only the legislation but also the culture of social safety has been improved. Given the economic and social development, the 5th Plenary Session of the 16th Central Committee of the Communist Party of China put safety in a strategic position; safety was as important as resources and the environment. Meanwhile, enterprises have also started given the due importance of caring for injured workers and community security, along with social progress and economic development. The 11th Five-Year Plan on mine safety, a programmatic document to guide coal production during the Plan, compiled by the State Administration of Work Safety, recommends strengthening the following measures in the implementation process: (a) carry out accountability for safe production in mines; (b) improve coal safety supervision; (c) increase safety investments in coal mines; (d) establish a comprehensive system of safety science and technology in coal mines; (e) improve safety regulations and systems for mine production; (f) upgrade mine safety; (g) promote the transformation and restructuring of coal enterprises and the construction of large-scale coal bases; (h) encourage the development of an in-house safety culture; (i) strengthen international exchanges and cooperation in mine safety; etc. Among these measures, the implementation of accountability culture for making production safe in coal enterprises is an important performance evaluation index of the cadres. The tightened law enforcement forces the coal industry managers to pay more attention to safety regulations and to understand them better. Increasing demands for investments in the safety culture of an enterprise directly affect managers’ perception of safety inputs. The improvement of safety climate strengthens the development of a safety culture and advocates safety, a human-oriented concept.

(2) Social safety environments have improved, as has the safety climate of society, and an increasing number of people are concerned about safety problems, mainly owing to the following aspects: (a) People′s living standards and demands for safety have been strengthened; (b) People′s cultural and educational level has been constantly improving; (c) The development of a safety culture has been intensified; (d) The development of a safety legal system has been playing a role in improving safety climate; (e) Safety culture has improved significantly. At present, there are several safety culture systems, such as the development, marketing, consulting, and service systems, the prevention of occupational diseases and the healthcare system, and the safety and occupational health information system in the press. In addition, there are related social groups, research institutes, and research teams in universities.

(3) Several types of media have strengthened public supervision. The media have greatly contributed to making mining accidents transparent, and safety problems have been prominent, together with the rapid development of network information. Under the influence of so many reports about coal mine accidents, senior managers in the industry may embrace the idea that the industry should enhance their safety awareness and improve safety climate. Meanwhile, enterprises can learn a lesson from authentic media reports about accident causes and accountability investigations to avoid similar accidents. As time passes, a good atmosphere will be formed, thus promoting further improvements in safety.

#### 4.1.3. Enhancement of a Staff′s Safety Demands

According to Maslow′s hierarchy of needs [31], safety requirements are only second to individuals′ physiological demand. After meeting the basic physical requirements, demands for safety are reflected intensively by the following: (a) The requirements for occupational safety in the work environment have been improved. When choosing employment in a given industry, people emphasize the work environment as well as wages; (b) Staff demands for safety training have increased. Safety training sessions, which were once regarded as a burden and as a way to avoid the control of higher authorities, are now the option in which most workers actively engage themselves of their own volition; (c) The integration of small mines and the collectivization of coal enterprises have improved the employment system. Previously, most small mines hired temporary and poorly qualified workers. Today, however, the overall quality is higher thanks to the reduction of temporary workers and the increase of formal employees. In addition, the training of formal workers is also relatively standardized; (d) Workers′ safety initiatives are emerging as safety demands increase and as senior managers are obliged to take workers’ safety demands into consideration, indirectly influencing their attitudes towards safety and awareness and ultimately improving overall safety climate.

#### 4.1.4. Scientific and Technological Progress in the Coal Industry

The scientific and technical aspects of the coal industry have gradually increased in recent years, and the use of new technologies and methods plays a positive role in promoting a safe environment. To cultivate talents and increase the scientific and technological base, investments are being made to modernize the coal industry. Today, enterprises′ competitiveness depends largely on the mechanization of the coal industry, the computerization of operation management, and the networking of safety monitoring. Scientific and technological progress in the coal industry mainly reflects the following changes: (a) Integrated technological innovation platforms by the collaboration of enterprises, universities, and research institutes have been developed; (b) Industry has seen many breakthrough technologies. Guided by national policies for science and technology development, a series of projects involving key technologies and some major coal technology equipment have been listed in national science and technology programs as well as in national priority development projects. These projects include “863” and “973” plans, high-tech industrialization, major equipment nationalization, etc.; (c) Information technology has been widely used in coal mines, making remarkable achievements, such as the integrated management information system on coal production, digital control of mine power systems, automatic unloading systems, and automatic control in coal mining; (d) The mechanization of coal mining is developing rapidly. The improvement of science and technology has mostly merged into a safety concept, and the application of new technologies strengthens the safety of equipment and overall working environment.

### 4.2. Causes of Partial Concepts in Decline

#### 4.2.1. Lack of Awareness of Safety Creating Economic Benefits

In spite of the increased investments in safety in recent years, managers′ understanding that safety creates economic benefits is still low. To change this situation, more evidence on “safety creating economic benefits” should be demonstrated, making people well aware of the relationship between safety and benefits; people with a poor understanding of safety investments believe that these are (as a rule) pure costs with no profits and that increasing these investments only raises enterprises′ expenses. On the contrary, safety investments are to be considered as special investments, whose benefits are not directly reflected in an increase in quantity and quality of products but ensure the normal and continuous running of the production process as a whole. In other words, safety investments can reduce losses due to accidents and losses that may be caused by the discontinuity in the production due to injured workers. Therefore, the economic benefits of safety investments are indirect: they are embodied in the guarantee of smooth production and cost savings from accident losses. Based on the principle of safety economics, the input–output ratio of preventive investments is much higher than that of accident repairs.

#### 4.2.2. Insufficient Communication on Safety and Experience

Communication among coal enterprises is insufficient because of their relatively isolated and inconvenient location. Besides, there is not enough attention paid to advanced management experience and scientific methods at home and abroad. Generally, enterprises build their own management modes on experience, often greatly affected by the turnover of managers and personnel. Therefore, it is necessary for the staff to strengthen communication on safety experience, advanced technologies, and management methods both inside and outside coal enterprises.

#### 4.2.3. Insufficient Attention Paid to a Safety Culture and Management Methods

Coal enterprises are generally more concerned about the development of external forms of safety, while ignoring the internal forms such as safety climate and management methods. The public coal enterprises can apply safety models, specific methods, and safety concepts promoted by state departments at different levels.

#### 4.2.4. Role of Safety Organizations Not Played Effectively

When turning to the role of safety organizations, the role of trade unions in accident prevention in coal enterprises is worth mentioning. According to the Trade Union Law of the PRC, trade unions have four basic functions: establishment, maintenance, participation, and education. Their first function is to safeguard workers′ legal rights. According to the seventh regulation of the Safety Production Law, trade unions shall require workers to participate in the democratic management and supervision of safety at work and safeguard their legal rights in safety production. The 23rd, 24th, and 25th regulations of the Mine Safety Act prescribe that (a) trade unions of mining enterprises should safeguard workers′ legal rights to safety and supervise mine safety; (b) if mining enterprise infringe laws and regulations on safety, trade unions are entitled to demand serious action by administrative or relevant departments; (c) when mining enterprises hold meetings on safety production, trade union representatives shall participate and be allowed to present their opinions and suggestions; (d) when trade unions find illegal situations, such as obviously hidden dangers and occupational hazards, they have the duty to propose solutions; moreover, when they find that certain situations endanger workers’ lives, they shall demand that the administrative department organize an evacuation from dangerous sites and take timely decisions to handle those situations. However, trade unions are not effectively playing their role at present in safety management and accident prevention. Repeated mine accidents are still due to the use of outdated equipment and technology, poor supervision, and loose enforcement of precautionary measures, along with the absence of trade unions in some enterprises.

## 5. Conclusions

The study of senior managers′ attitudes towards safety in the Chinese coal industry reveals that three concepts were at a very high level in 2014. It is thus evident that managers are paying more attention to strengthening safety management systems, safety professional institutions, safety working groups, safety training, etc. In addition, there are six concepts at a relatively high level. This shows that, apart from attributing importance to management and systems, senior managers also realize the importance of safety staff selection, accident prevention, and safety-creating benefits. However, senior managers pay little attention to certain safety concepts, such as safety investment, safety regulation, and safety methods.

Analyses of changing trends over the period of five years from 2009 to 2014 revealed the causes of improvements in some safety concepts, such as the improvement in social and legal environments, the improvement in the social safety culture environments, the enhancement of the staff′s safety demands, and scientific and technological progress in the coal industry. Because of the safety climate being closely linked to safety conditions, further efforts should be made to strengthen the above aspects for the promotion of safer climate.

Analyses of changing trends also showed the reasons for the decline of four safety concepts, further suggesting the following: (a) At present, the evidence of safety-creating economic benefits should be strengthened to improve the understanding of the nature of safety investments; (b) Safety experience, both inside and outside the coal, and other, industries, at home and abroad, should be stressed through publicity, campaigns of information, and stronger communications so as to improve the level of safety management; (c) More attention should be paid to incentives in safety management to improve employee participation; (d) Safety organizations, especially unions, should include accident prevention as the main focus in their agenda.

## Figures and Tables

**Table 1:** Fifteen important safety concept indexes.

Safety Concept Index	Description
A perception of the relative importance of safety	A deep understanding of the Chinese safety production policy “Safety being First, Mainly for Prevention, And to Control Comprehensively” is important, as it reflects senior managers’ opinions on the relationship among safety, profit, and production, as well as the degree of care in the policy of putting safety and prevention first.
An understanding that casualties are preventable	“Zero accidents” is a common view of enterprises with good safety management at home and abroad and is achievable. This principle means that, if employees can realize that all accidents can be prevented, they will pay attention to safety details and make every effort to prevent accidents.
An understanding that safety creates economic benefits	As long as managers are able to recognize the economic benefits (indirectly and in the long term), they will reasonably invest in safety. That safety creates economic benefits mainly manifests in loss and profit.
The degree of safety merged into enterprise management	Before starting any project, safety should be considered first. According to the pyramid principle of safety, a 1 point of safety at the design stage is equivalent to 10 points of safety at the processing and manufacturing stages, and to 1000 points at the operation and production phases.
The opinion that safety mainly depends on safety awareness	Having safety awareness means having the ability to find and deal with hazards timely. Not being aware of safety is the greatest hazard in work situations and is currently an important cause of accidents in production. In order to promote safety behaviors and a production facility’s safety conditions, safety awareness must first be ensured.
An understanding the responsibility system of work safety	General responsibility standards should be aligned along those of government and enterprises. The government is the regulatory body of safety production, which requires the establishment of a safety production responsibility system for government administrative leaders, whereas enterprises are the main body of safety production, which also require the establishment of a safety production legal responsibility system.
An opinion regarding safety investment	Safety investment refers to all costs of accident prevention, which mainly includes safety devices, safety training, safety activities, safety reward funds, and so on. Safety investment is essential to guarantee safe working conditions, and each enterprise should increase the money it spends on safety, in addition to that mandated by the State.
The role of safety regulation	Workers′ safety and health should be legally safeguarded. Currently, there are over 30 special laws and administrative regulations on safety production in China. Enterprises need to do better than the requirements of safety laws and regulations in order to prevent major accidents.
The degree to which managers are responsible for safety	Managers′ behaviors have been an important reference index in safety production, and enterprises should emphasize their responsibility for safety. They are the promoters of safety culture, the supporters of safety rules and regulations, the organizers of the implementation of the responsibility system of safety production, and the decision-makers with respect to enterprise safety resource investments.
Demands for safety training	Safety training is an important way for the Chinese government to strengthen safety production. Safety training is not only a legal obligation but also a positive safety investment for enterprises to reduce accident losses. In addition, demands for safety training also reflect the level of individual safety awareness.
An understanding of the role of safety management systems	An effective safety management system can enhance a staff′s ability to deal with safety problems, to deal with emergency situations, to control accidents, and to effectively minimize occupational risks.
A mastery of safety methods	There are many methods in safety management. Enterprises should not only be familiar with their own safety methods but also adopt those of other companies. Different management methods have different emphases, applications, and implementation effects. Managers should master all safety methods and carry out effective safety management.
Awareness of safety performance and human resources	Safety performance assessment and improvement are important to link enterprise performance management and safety management. Enterprises should make the performance evaluation index and evaluation method conducive to accident prevention. This makes employees aware that safety performance is directly related to their own benefit, thus paying more attention to safety. Effective appointments and the rational use of human resources based on matched talents and posts are also important. The maximum safety performance can be achieved only when staff can meet the work requirement and the best fit for the job.
A recognition of the work of the safety department	Working as an assistant for enterprise managers, the safety department is responsible for making a production facility safe by developing excellent guidelines, by developing and implementing regulations and standards on safe production and labor protection, by checking the quality of the department′s safety systems and regulations, and by assisting in adopting correct measures for protecting workers from fatal and occupational injuries and diseases to make production process run smoothly.
Emergency response capability	When accidents happen, the accident loss depends on the accident emergency ability of the enterprise and its staff. It is obvious that accidents can be avoided, but sometimes they may occur suddenly and unexpectedly. Personnel therefore should be able to deal with an emergency situation. In order to adopt effective and timely measures when an emergency occurs, the enterprise should produce detailed emergency plans.

**Table 2:** Comparative statistics analysis of 15 evaluation concepts in 2009 and 2014.

Question Number	Specific Questions	Second Stage, 2014 (Mean ± Standard Deviation)	First Stage, 2009 (Mean ± Standard Deviation)	*t*
1	Perception of the relative importance of safety	3.8986 ± 0.77638	3.6625 ± 1.60650	0.968
2	Understanding that casualties are preventable	4.2319 ± 1.20395	3.7262 ± 0.84099	1.554
3	Understanding that safety creates economic benefits	4.0435 ± 0.94266	4.4643 ± 0.73544	−3.494
4	Degree of safety merged into enterprise management	4.9275 ± 0.31129	4.8571 ± 0.44296	1.388
5	Opinion that safety mainly depends on safety awareness	4.2029 ± 1.32444	4.5422 ± 1.08539	−1.969
6	Understanding the responsibility system of work safety	3.9638 ± 1.06989	3.6786 ± 0.92046	2.028
7	Opinion on safety investment	3.2059 ± 1.44077	2.2024 ± 0.96667	5.645
8	Role of safety regulation	3.5036 ± 1.16394	2.1310 ± 0.69038	9.799
9	Managers′ responsibility degree for safety	3.2993 ± 1.03870	4.6429 ± 0.83078	−10.045
10	Demands for safety training	4.8551 ± 0.47646	4.9048 ± 0.36797	−0.818
11	Understanding the role of safety management systems	4.1014 ± 0.55750	3.4048 ± 1.60661	4.660
12	Mastery of safety methods	3.5556 ± 1.3082	4.9157 ± 0.27958	−9.334
13	Awareness of safety performance and human resources	4.3206 ± 0.91372	3.0357 ± 1.35723	8.297
14	Recognition of the work of the safety department	4.8939 ± 0.44990	3.2500 ± 1.45515	19.485
15	Emergency response capability	4.0876 ± 1.20339	3.2619 ± 1.21357	4.935

Notes: Here, when *t* > 1, significance is increased; when *t* < −1, significance is decreased; when |*t*| < 1, there is no significant effect; all data are 2014 vs. 2009.
